# A Novel Stent Coated with Antibodies to Endoglin Inhibits Neointimal Formation of Porcine Coronary Arteries

**DOI:** 10.1155/2014/428619

**Published:** 2014-05-04

**Authors:** Song Cui, Jing-Hua Liu, Xian-Tao Song, Guo-Lin Ma, Ben-Jun Du, Shu-Zheng Lv, Li-Jun Meng, Quan-Sheng Gao, Kefeng Li

**Affiliations:** ^1^The Key Laboratory of Remodeling-Related Cardiovascular Diseases, Department of Cardiology, Anzhen Hospital Affiliated to Capital Medical University, Beijing 100029, China; ^2^Department of Radiology, China-Japan Friendship Hospital, Beijing 100029, China; ^3^Southern Hospital Affiliated to Southern Medical University, Guangzhou 510515, China; ^4^Department of Cardiology, Binzhou Central Hospital, Binzhou 251700, China; ^5^Laboratory of the Animal Center, Academy of Military Medical Sciences, No. 27 Taiping Road, Haidian District, Beijing 100850, China; ^6^School of Medicine, University of California, San Diego, CA 92103, USA

## Abstract

Endoglin/CD105 is an accessory protein of the transforming growth factor-*β* receptor system that plays a critical role in proliferation of endothelial cells and neovasculature. Here, we aimed to assess the effect of novel stents coated with antibodies to endoglin (ENDs) on coronary neointima formation. Thirty ENDs, thirty sirolimus-eluting stents (SESs), and thirty bare metal stents (BMSs) were randomly assigned and placed in the coronary arteries in 30 juvenile pigs. Histomorphometric analysis and scanning electron microscopy were performed after stent implantation. Our results showed that after 7 days, there was no difference in the neointimal area and percent area stenosis in ENDs compared with SMSs or BMSs. After 14 days, the neointima area and percent area stenosis in ENDs were markedly decreased than those in BMSs or SESs (*P* < 0.05). Moreover, the percentage of reendothelialization was significantly higher in ENDs than that in SESs or BMSs (*P* < 0.01) at 7 and 14 days. The artery injury and the inflammation scores were similar in all groups at 7 and 14 days. In conclusion, our results demonstrated for the first time to our knowledge that endoglin antibody-coated stents can markedly reduce restenosis by enhancing reendothelialization in the porcine model and potentially offer a new approach to prevent restenosis.

## 1. Introduction

Angioplasty is now the most common procedure performed to widen narrowed or blocked coronary arteries. The major complication of angioplasty is in-stent restenosis (ISR) [1]. Coronary artery stent implantation has been used for years to dramatically reduce the incidence of ISR and to improve the flow of blood to the heart tissue [1]. There are two basic kinds of stents: bare-metal stents (BMSc) and drug-eluting stents (DESs). The BMSc are metal stents with no special coating. As the artery heals, tissue growth over the stents eventually leads to reblockage. In contrast, the invention of the DESs that are coated with medication can reduce this risk [1, 2].

Restenosis is mainly characterized by intimal hyperplasia and vessel remodeling and is believed to be due to dysfunctional arterial healing involving primarily platelet aggregation and hyperplastic inflammatory pathways [3]. It has been shown that a functionally intact endothelium is a prerequisite for the inhibition of neointimal growth after percutaneous coronary intervention (PCI) [4] and that endothelial progenitor cells (EPCs) may play a major role in reendothelialization (RE) and inhibition of stent neointimal formation [5]. Indeed, infusion of EPCs after vascular injury and their mobilization and incorporation after statin treatment significantly inhibit neointimal growth [5, 6]. Recently, clinical studies suggested that DESs significantly reduce neointimal growth and revascularization rates compared with BMSs but delay reendothelialization and, in some studies, appear to be accompanied by a higher prevalence of stent thrombosis [7–9]. However, recent studies with antibody-coated stents had shown improved stent endothelialization as well as feasibility and safety in the clinical setting [10–12].

Endoglin (also known as CD105) is a homodimeric membrane glycoprotein that binds transforming growth factor (TGF)-*β*1 and -*β*3 isoforms in human endothelial cells (ECs) [13–15]. Emerging evidence has shown that endoglin expression is mainly restricted to vascular endothelial and stromal cells, while it is detectable on activated monocytes, macrophages, and other cells [16–18]. Studies on the cellular and tissue distribution of endoglin suggest its profound functional involvement in angiogenesis, vascular development, and homeostasis [19]. Interestingly, endoglin is strongly expressed in the angiogenic vasculature of solid tumors, and the level of endoglin positively correlates with the extent of endothelial cell proliferation [19]. Several studies have demonstrated that vascular targeting by antiendoglin antibody is a useful and safe procedure for tumor imaging and treatment in vitro and in vivo [19]. However, it is unclear whether endoglin antibody coated stents could reduce neointimal hyperplasia.

In this study, using the healthy coronary porcine model, we evaluated the differences in coronary neointimal hyperplasia and reendothelialization between endoglin antibody-coated stents (ENDs), sirolimus drug-eluting stents (SESs), and bare-metal stents (BMSs).

## 2. Materials and Methods

### 2.1. Animal Model

Thirty 4-to-5-month-old juvenile pigs with the weight of 25–35 kg were purchased from the Agricultural University Experimental Animal Center, China. Thirty stainless steel stents coated with murine monoclonal anti-human endoglin antibody (ENDs) (Beijing Lepu Medical Technology limited corporation, China), thirty sirolimus-eluting stents (SESs) (purchased from Johnson & Johnson, USA), and thirty bare metal stents (BMSs) (purchased from Abbott, USA) were randomly assigned and placed in the left anterior descending, circumflex, or right coronary arteries (one stent per artery) of 30 pigs. The stents were implanted using an incomplete factorial design, thus allowing intraindividual and interindividual comparisons, and the pigs were randomly assigned to these treatment modalities. Pigs were maintained on 75 mg clopidogrel and 100 mg aspirin per day and sacrificed after 7 days and 14 days, respectively. Thirty stents of each type were then subjected to histology and scanning electron microscopy experiments or angiography.

### 2.2. Study Procedures

The study protocol was reviewed and approved by the Institutional Animal Care and Research Committee of Capital Medical University, China. All animals received humane care in compliance with the Animal Welfare Act. All domestic pigs were treated according to local standard interventional techniques. Specifically, the decision of predilation or direct stent was at the investigator's discretion, and postdilation was performed as required to ensure that the residual stenosis was less than 20% by visual assessment, with a TIMI flow grade rate. All domestic pigs were scheduled for a follow-up at 7 days and 14 days following the implantation procedure to assess the anginal status and the occurrence of major adverse cardiac and cerebrovascular events (MACCE).

The cross-sectional area of stenosis was determined using intravascular ultrasound (IUVS). Briefly, prior to IVUS examination, low dose of heparin (100 U/kg) was administrated to the animals. IVUS examination was performed on a Jomed 30 MHz catheter with 2.9 Fr phased array probe (Invision Gold; Jomed, Sweden). IVUS imaging was performed during the motorized pullback (0.5 mm/s) of the imaging catheter. The images were recorded on the compact disc for offline analysis. Cross sections obtained with ultrasound were matched with the corresponding histologic sections. The percentage of cross-sectional area of stenosis was calculated by the following equation:

(1) $$\begin{array}{c} \left[\frac{\left(\text{external}\;\text{elastic}\;\text{membrane}\;\text{area}-\text{lumen}\;\text{area}\right)}{\text{external}\;\text{elastic}\;\text{membrane}\;\text{area}}\right]*100\%. \end{array}$$

### 2.3. Histomorphometric Analysis

Seven or 14 days after stent implantation, animals were euthanized using intravenous injection of pentobarbital euthanasia solution (100 mg/kg), and the stented coronary arteries were harvested. The arteries were sectioned into 3 to 5 mm segments from the proximal, middle, and distal part of the stents, fixed in 4% formalin for 48 h, and embedded in paraffin. Serial 5 *μ*m sections from the distal part of the stents were subjected to haematoxylin and eosin (H&E) staining as described by Li et al. [20]. The lumen area (LA) as well as the area circumscribed by the internal elastic lamina (IEL area) was measured using a computer-assisted digital system (Image-Pro Plus, Media Cybernetics, Silver Spring, MD, USA). Neointima area (NA) was defined as the IEL area minus the LA (IEL area-LA). The statistical analysis for treatment effects was conducted based on the average of all sections per stent.

### 2.4. Scanning Electron Microscopy (SEM)

Intact stented arterial segments were opened longitudinally, flattened, and fixed in 1.6% glutaraldehyde before being dehydrated, dried with liquid CO_2_, and coated with gold. The specimens were visualized using a Hitachi Model 3600N SEM (Tokyo, Japan) to determine the percent area reendothelialization (RE) compared with the total luminal surface area. For each specimen, five separate SEM photomicrographs were taken at ×400 magnification and the RE area for each artery was represented by the sum of the data for the five photographed subareas [21].

Stent endothelialization was assessed using the method described by a previous study [22]. Stent endothelialization was scored by the extent of the circumference of the arterial lumen covered by endothelium: 1 = 25%; 2 = 25–75%; and 3 = 75–100% coverage, and data were expressed as average of six independent scores (*n* = 6).

### 2.5. Evaluation of Arterial Injury and Inflammation Scores

The severity of arterial injury was scored as previously described by Schwartz et al. [23]: 0 means no injury, 1 means break in the internal elastic membrane, 2 means perforation of the media, and 3 means perforation of the external elastic membrane to the adventitia. The inflammation score for each individual strut was graded according to the following criteria: 0 means no inflammatory cells surrounding the strut, 1 means light, noncircumferential lymphohistiocytic infiltrate surrounding strut, 2 means localized, moderate-to-dense cellular aggregate surrounding the strut noncircumferentially, and 3 means circumferential dense lymphohistiocytic cell infiltration of the strut. Arterial injury and inflammation scores for each cross section were calculated by dividing the sum of the individual injury and inflammation scores by the total number of struts at the examined section, as previously described [23, 24].

### 2.6. Statistical Analysis

Statistical analysis was performed with the aid of the commercially available software (SPSS Version 11, Chicago, IL, USA). The data were presented as mean ± SD. Student-Newman-Keuls was used for the comparison of inflammatory cell counts normalized to injury score of the two stent groups. Analysis of variance (ANOVA) was used for comparisons of the three stent groups. Significance was established at the 95% confidence level (*P* < 0.05).

## 3. Results

### 3.1. Procedural Characteristics

A total of 90 stents including thirty SESs, thirty BMSs, and thirty ENDs, were randomly placed in the proximal left anterior descending, proximal circumflex, and proximal right coronary artery for thirty pigs. No death was observed during this study. Quantitative coronary angiography before and after stent implantation indicated that stent-to-artery ratio was 1.1 to 1.2 for all 90 stented arteries. There was no significant difference in stent-to-artery ratio among three stent groups (data not shown).

### 3.2. Histomorphometric Analysis

All vessels were examined by histologyand angiography at two time points. Stent malapposition was not detectable in histologic specimens or by intravascular ultrasound examination after stent implantation (data now shown).

**Figure 1:**
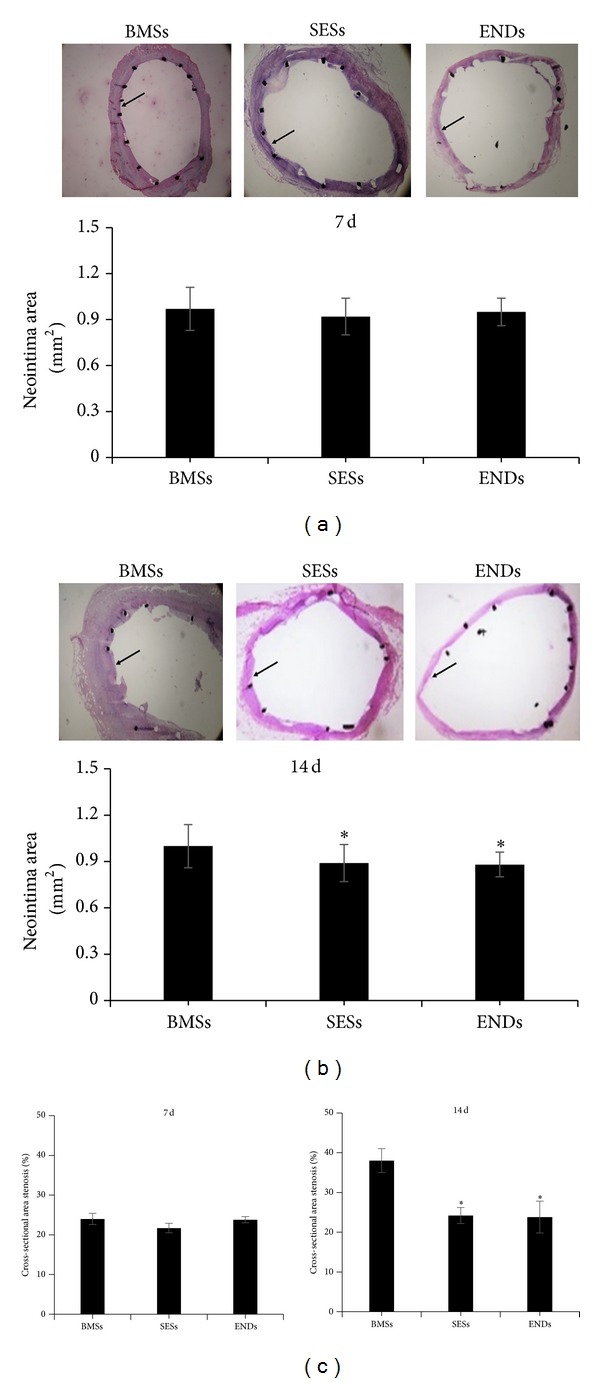
Histomorphometric analysis. Representative photos of haematoxylin-and-eosin-stained cross sections of arteries at 7 ((a), top panels) and 14 days ((b), top panels) after stent implantation (magnification, ×40). Bar graphs show mean neointima area ((a) and (b), bottom panels) and percent area stenosis (c) of stented arteries. Data represent the mean ± SEM (*n* = 15). *P < 0.05 versus BMSs. BMSs: bare metal stents; SESs: sirolimus-eluting stents; and ENDs: endoglin antibody. Arrow indicated neointima.

Seven days after stent implantation, mean neointima area was 0.95 ± 0.09 mm^2^, 0.92 ± 0.12 mm^2^, and 0.97 ± 0.14 mm^2^ for ENDs, SESs, and BMSs, respectively (*P* > 0.05). The percent area stenosis was 23.80 ± 3.10%, 21.70 ± 2.30%, and 24.00 ± 3.10% for ENDs, SESs, and BMSs, respectively (*P* > 0.05). There were no differences in the neointima area and percent area stenosis among three stent groups (Figure 1(a)).

**Figure 2:**
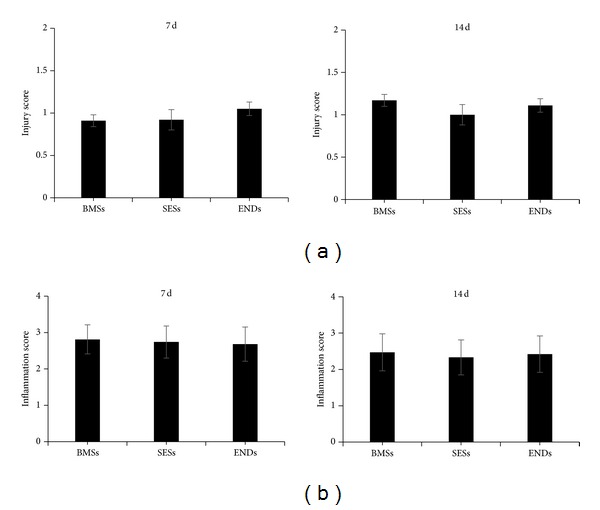
Quantification of artery injury and infiltration scores around the stent struts at 7 (a) and 14 days (b) after stent implantation. Data represent the mean ± SEM (*n* = 15).

Fourteen days after stent implantation, mean neointima area of ENDs or SESs was significantly lower (0.9 ± 0.08 mm^2^ and 0.95 ± 0.12 mm^2^) as compared with BMSs (1.25 ± 0.14 mm^2^) (*P* < 0.05, Figure 1(b)). The corresponding percent area stenosis was 23.80 ± 4.00%, 24.20 ± 2.20%, and 38.0 ± 3.20% for ENDs, SESs, and BMSs, respectively. Importantly, percent area stenosis in ENDs and SESs was less than that in BMSs (*P* < 0.01 for ENDs and SESs versus BMSs), whereas no differences in mean neointima area and percent area stenosis were observed between ENDs and SESs (*P* > 0.05, Figure 1(b)). In addition, arterial injury and inflammation scores were similar in all three stent groups at 7 and 14 days (*P* > 0.05, Figure 2).

### 3.3. Evaluation of Stent Reendothelialization

**Figure 3:**
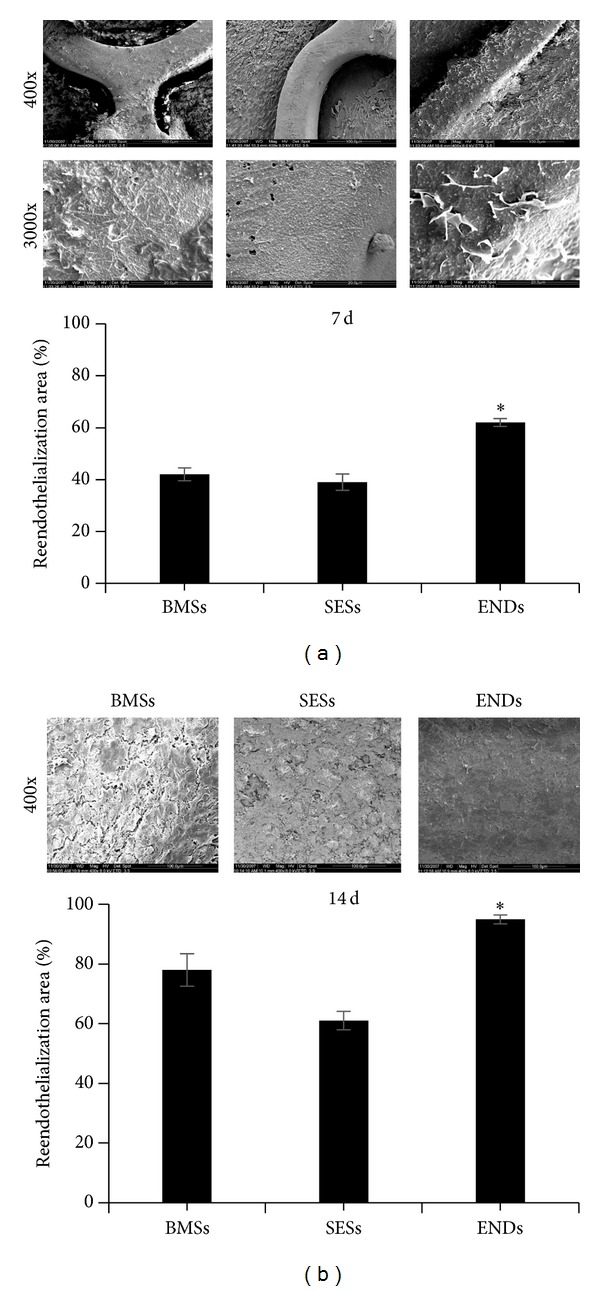
Reendothelialization of arteries assessed by SEM at 7 ((a), top) and 14 days ((b), top) after stent implantation. SEMs images are the intact stented arterial segments. Bar graphs indicate percentage of re-endothelialized area of total stented area ((a) and (b), bottom). Data represent the mean ± SEM (*n* = 15). The scale bar represents 100 *μ*m (top panels) and 20 *μ*m (bottom panels) in (a) and 500 *μ*m in (b). *P < 0.01 versus BMSs or SESs.

To determine the effect of coating stents with antibodies to endoglin on reendothelialization, stents coverage by endothelial cells was evaluated by SEM. Seven days after stent implantation, there was a significantly higher extent of endothelial coverage above struts in ENDs (60.63 ± 5.60%) or BMSs (41.30 ± 6.80%) compared with SESs (35.82 ± 4.95%) (*P* < 0.01, Figure 3(a)). After fourteen days, the RE areas for all stents were higher, but ENDs (94.64 ± 6.90%) showed the greatest endothelialization compared with BMSs (75.61 ± 7.87%) or SESs (65.21 ± 8.39%) (*P* < 0.01, Figure 3(b)). No significant differences were observed in RE between BMSs and SESs at 7 and 14 days (*P* > 0.05) (Figures 3(a) and 3(b)).

## 4. Discussion

Drug eluting stents (DES) have been successfully demonstrated to be effective in reducing in-stent restenosis and neointimal hyperplasia [25, 26]. However, several recent studies reported that DESs could potentially increase the risk of late stent thrombosis [27, 28]. Regarding the next generation stents, great efforts have been placed on facilitating endothelialization using new approaches such as biocompatible/biodegradable polymers, polymer-free drug eluting, and the prohealing endothelial progenitor cells capture. Instead of inhibiting vascular smooth muscle cell growth using drugs, the prohealing approach has focused on endothelialization on the stent surface. The Genous TM bioengineered R stent (Orbusneich, USA) coated with anti-CD34 antibodies through a biocompatible matrix was developed. The antibodies helped capture endothelial progenitor cells (EPCs) from circulation, thus facilitating stent endothelialization [29]. Clinical trials have indicated that this technology significantly reduced the delayed stent thrombosis and was safe [30].

In the present study, we sought to determine whether endoglin antibody-coated stent is beneficial for arterial healing after stent implantation. The principal findings are that ENDs can significantly reduce the extent of neointimal hyperplasia and accelerate stent reendothelialization compared with the SESs or BMSs.

Increasing evidence has demonstrated that current used drug-eluting stents (DESs) such as sirolimus and paclitaxel-eluting stents significantly reduced the incidence of restenosis [1]. However, DESs with nonselective drugs inhibit smooth muscle cell proliferation but also delay or prevent the proliferation of endothelial cells to cover stents [1]. Moreover, these stents have been known to cause restenosis and lead to blood clots. In addition, patients who receive DESs must take blood-thinning drugs that have potential side effects, including rashes and bleeding [1]. Because of these disadvantages, how to combat vascular restenosis and speed up the repair of damaged intima is a new and promising research direction. Recently, a new type of device, the antibody-coated stents, has been designed to accelerate healing of vascular damage. Several studies have demonstrated that antibody-coated stents such as CD34 antibody-coated stents drastically reduce rates of restenosis and thrombosis [10–12, 24].

Endoglin is an accessory protein of the transforming growth factor-*β* (TGF-*β*) receptor family [31]. Although endoglin protein itself lacks kinase activity, it can be a regulator of TGF-*β* signaling to mediate a variety of processes, including cell proliferation, migration, and adhesion. Endoglin is known to inhibit the biological effects of TGF-*β* on synthesis of fibronectin, cell adhesion, platelet-endothelial cell adhesion molecule-1 phosphorylation, and the same type of aggregation that endoglin functions as an auxiliary receptor which contributes to the complex regulation of TGF-13 responses [32]. In endothelial cells, endoglin expression is upregulated by hypoxia, TGF-*β* stimulation, and irradiation in vitro, whereas endoglin is downregulated by tumor necrosis factor-*α* [33]. Importantly, endoglin has a role in the development of the cardiovascular system and in vascular remodeling [19] and is emerging as a marker of activated endothelial cells, and its vascular expression is limited to proliferating cells. Based on antigen-antibody binding principle, we hypothesized that endoglin antibody-coated stents (ENDs) may automatically attract and capture circulating activated endothelial cells, leading to increased stent reendothelialization. Indeed, our results suggested that ENDs markedly inhibit neointimal hyperplasia at 14 days after stent implantation and promote reendothelialization at 7 and 14 days after stent implantation compared with SESs or BMSs.

Restenosis is mainly characterized by intimal hyperplasia and vessel remodeling. It is a combined result of a biological response and mechanical reaction to percutaneous coronary intervention (PCI). However, angioplasty can irritate or damage arterial walls and trigger smooth muscle cells to neointimal hyperplasia during the healing process. Scar tissue forms and then bulges into the arterial lumen, thus narrowing the vessel's internal diameter [1]. Endothelial denudation is considered to be the primary injury after balloon angioplasty and stent implantation. When larger areas are denuded or endothelial recovery is delayed, a higher degree of intimal thickening occurs [34]. Reendothelialization after stent implantation and vascular injury is a critical step in the process of vascular healing. Several studies have demonstrated the importance of adjacent recruitment of ECs or blood-derived EPCs for the formation of new endothelium after vascular injury [35]. The early establishment of a functional endothelial layer after vascular injury has been shown to assist in the prevention of neointimal proliferation and thrombus formation. A previous study showed that EPCs can be used as a therapeutic approach to accelerate reendothelialization after myocardial injury [36]. Treatment with statins and the inhibition of promigratory factors have implicated the recruitment of EPCs in enhancing endothelial recovery and reducing neointima [37]. However, the mechanisms underlying EPC recruitment (homing) to the site of injury remain to be elucidated. Abciximab is a monoclonal antibody that blocks platelet glycoprotein IIb/IIIa receptor. Recently, the stents coated with antibodies including anti-CD34 and anti-abciximab can speed healing of angioplasty-induced vascular damage by recruiting EPCs to the site of vessel injury [10–12, 31]. EPCs proliferate and re-endothelialize more rapidly to reduce the risk of restenosis and thrombosis. In this study, our data indicated that ENDs markedly promote the coverage of endothelial cells on the surface of stents at 7 and 14 days. However, it is unclear whether local delivery of antibody endoglin-coated stents is capable of mediating the attraction of EPCs, thereby enhancing coronary reendothelialization.

## 5. Conclusion

The present study showed that endoglin antibody-coated stents could promote the endothelial cell coverage and reduce restenosis. Thus, the present novel findings may provide a promising target for stent development to accelerate healing and reduce restenosis after stent implantation.
